# Hybridization Chain Reaction-Enhanced Ultrasensitive Electrochemical Analysis of miRNAs with a Silver Nano-Reporter on a Gold Nanostructured Electrode Array

**DOI:** 10.3390/jfb16030098

**Published:** 2025-03-12

**Authors:** Bin Wang, Huiqiang Ma, Mingxing Zhou, Xian Huang, Ying Gan, Hong Yang

**Affiliations:** 1 Intensive Care Unit, Department of Emergency Medicine, The Second Hospital of Tianjin Medical University, Tianjin 300211, China; doctorwb2019@163.com; 2 Department of Pharmacology and Tianjin Key Laboratory of Inflammation Biology, The Province and Ministry Co-Sponsored Collaborative Innovation Center for Medical Epigenetics, School of Basic Medical Sciences, Tianjin Medical University, No. 22 Qixiangtai Road, Heping District, Tianjin 300070, China; 3 Department of Biomedical Engineering, Tianjin University, 92 Weijin Road, Tianjin 300072, China

**Keywords:** electrochemical detection, HCR, nanostructured electrode, miRNAs, silver nanoparticles

## Abstract

Abnormal expression of miRNAs is associated with the occurrence and progression of cancer and other diseases, making miRNAs essential biomarkers for disease diagnosis and prognosis. However, the intrinsic properties of miRNAs, such as short length, low abundance, and high sequence homology, represent great challenges for fast and accurate miRNA detection in clinics. Herein, we developed a novel hybridization chain reaction (HCR)-based electrochemical miRNAs chip (e-miRchip), featured with gold nanostructured electrodes (GNEs) and silver nanoparticle reporters (AgNRs), for sensitive and multiplexed miRNA detection. AgNRs were synthesized and applied on the e-miRchip to generate strong redox signals in the presence of miRNA. The stem–loop capture probe was covalently immobilized on the GNEs, and was opened upon miRNA hybridization to consequently trigger the HCR for signal amplification. The multiple long-repeated DNA helix generated by HCR provides the binding sites for the AgNRs, contributing to the amplification of the electrochemical signals of miRNA hybridization. To optimize the detection sensitivity, GNEs with three distinct structures were electroplated, in which flower-like GNEs were found to be the best electrode morphology for miRNAs analysis. Under optimal conditions, the HCR-based e-miRchip showed an excellent detection performance with an LOD of 0.9 fM and a linear detection range from 1 fM to 10 pM. Moreover, this HCR-based e-miRchip platform was able to effectively distinguish miRNAs from the one- or two-base mismatches. This HCR-based e-miRchip holds great potential as a highly efficient and promising miRNA detection platform for the diagnosis and prognosis of cancer and other diseases in the future.

## 1. Introduction

MicroRNAs (miRNAs) are an endogenous small non-coding RNA with a length of approximately 19–25 nucleotides that can regulate gene expression though binding and silencing its target genes [1,2]. Studies show that miRNAs can act as oncogenes or tumor suppressor genes to influence diverse biological processes, and thus play a critical role in the occurrence and development of cancer, making them important tumor biomarkers [3,4,5]. However, the intrinsic properties of miRNAs, such as the short sequence, low abundance and high sequence homology represent great challenges for their analysis in the early diagnosis of tumors.

The current gold standard technology for nuclei acid analysis is RT-qPCR (real-time quantitative polymerase chain reaction), which utilizes a polymerase chain reaction-based amplification strategy combined with real-time fluorescence quantification to detect miRNAs with excellent sensitivity and accuracy. However, this technique often requires expensive thermal cycling machines for complex temperature control, thus limiting its application scenarios [6,7,8]. To overcome this challenge, researchers have endeavored to develop various electrochemical detection methods, due to their inherent advantages such as simplicity, rapidness, sensitivity, low cost and ease of multiplexing [9,10] in miRNAs analysis.

To enhance the miRNA readout, numerous nucleic acid amplification strategies were developed, including rolling circle amplification (RCA) [11], strand displacement amplification (SDA) [12], loop-mediated isothermal amplification (LAMP) [13], hybridization chain reaction (HCR) [14] and so on [15]. Among them, HCR technology is considered one of the most attractive strategies due to its enzyme-free features, high amplification efficiency and short reaction time. For example, Fan’s group designed an electrochemical biosensor combining the DNA tetrahedral nanostructure with HCR for miRNA-122b detection with a limit of detection (LOD) of 10 aM [16]. Yang’s group proposed a signal amplification strategy based on HCR, target-assisted polymerase nicking reaction (TAPNR) and AgNCs (Ag nanoclusters) to detect miRNA-199 as low as 0.64 fM [17]. In addition, Miao’s group also achieved detection of miRNA-21 (miR-21) using HCR and AgNPs (silver nanoparticles) based on the DNA tetrahedral nanostructure [18]. These examples suggest that the HCR-based electrochemical amplification strategy is efficient and reliable for miRNA sensing. However, all of these studies utilized a bulk gold electrode, which is unable to perform a multiplexed miRNA analysis.

In the attempts to enhance the performance of the electrochemical detection platform for miRNA analysis, various microelectrodes were fabricated due to their advantages in fast mass transfer, small RC time constant and excellent spatiotemporal resolution in comparison with the macroelectrode [19]. In addition, the planar microelectrodes could be further formed into three-dimensional nanostructured electrodes through electrodeposition of metals to increase the surface area of the electrode and improve the target recognition efficiency [20,21]. For example, Su’s group found that the sensitivity for electrochemical miRNA analysis was critically dependent on the morphology of the nanostructured array, in which the “dendritic” gold nanostructured array showed the lowest LOD (100 aM) for miR-21 detection compared with that of the flower-like and spherical gold nanostructured array [22]. Furthermore, Soleymani’s group deposited a single nanostructured gold and palladium electrode on a microporous electrode and further verified that only highly branched electrodes with fine structuring could lead to an attomolar sensitivity [23]. We previously proposed an e-miRchip utilizing flower-like gold nanostructured electrodes (GNEs) combined with the silver nanoparticle reporters (AgNRs) to profile miR-21 [12]. However, whether the flower-like GNEs would be the best morphology for this AgNRs-based miRNA detection platform remains to be clarified.

Herein, we aimed to develop a new signal amplification strategy based on HCR and AgNRs on an e-miRchip electroplated with nanostructured microelectrodes to achieve sensitive and multiplexed miRNA detection. Considering the importance of the electrode morphology on electrochemical detection of miRNAs, we fabricated three morphologies of GNEs and found that the compact flower-like nanostructured electrodes were able to achieve the best detection performance for miR-21 with a low LOD of 0.9 fM. Moreover, the excellent selectivity of the HCR and AgNRs-based e-miRchip for miR-21 detection was also verified. We believe that this platform represents a very promising gene detection platform and holds great potential in analyzing miRNAs in clinical samples for early diagnosis and real-time monitoring of cancer.

## 2. Experiments and Methods

### 2.1. Chemicals and Reagents

All reagents used were analytical grade unless otherwise noted. Sodium citrate (C_6_H_5_Na_3_O_7_), phosphate buffered saline (PBS), and tannic acid (C_76_H_52_O_46_) were purchased from BBI (Shanghai, China). Silver nitrate (AgNO_3_) was purchased from Tianjin Yingda Rare Chemical Reagent Factory (Tianjin, China). Tris(hydroxymethyl)aminomethane, NaCl, and MgCl_2_ were purchased from Sangon Biotech (Shanghai, China). Diethyl pyrophosphate (DEPC), potassium chloride (KCl), and β-mercaptohexanol (MCH) and sodium borohydride (NaBH_4_) were purchased from Sigma-Aldrich (St. Louis, MO, USA). Chloroauric acid (HAuCl_4_·4H_2_O) was purchased from Yuanye (Shanghai, China). Ethanol, acetone and isopropanol were obtained from Sinopharm Chemical Reagent (Shanghai, China). Hydrogen peroxide (H_2_O_2_), hydrochloric acid (HCl, 35–37%), nitric acid (HNO_3_, 65–68%), and sulfuric acid (H_2_SO_4_, 98%) were purchased from Sinopharm (Beijing, China). The ultrapure water with a resistivity of 18 MΩ·cm was obtained using a Millipore Milli-Q^TM^ water purification system (Millipore Corporation, Milford, MA, USA).

The nucleic acids used were synthesized by Sangon Biotech (Shanghai, China) and the specific sequences are shown below (Table 1).

### 2.2. Fabrication of HCR-Based e-miRchip

The chip was fabricated based on the microfabrication technology [12]. Briefly, the chromium (Cr) layer as an adhesive layer (5 nm) and gold layer (200 nm) were first deposited on a glass substrate by magnetron sputtering, respectively. Subsequently, AZ4620 photoresist was spin-coated onto the gold layer surface and etched through photolithography to obtain the gold sensing area, pads and the leads. Finally, a polyimide (PI) insulating layer (1 μm) was spin-coated on the entire chip followed by photolithography to obtain the gold aperture with a diameter of 10 μm.

### 2.3. Synthesis of AgNPs and AgNRs

Three different sizes of AgNPs were synthesized. For the synthesis of AgNPs with a diameter of 10 nm (AgNP_10_), the method established in the literature was adopted [24]. AgNO_3_ (1 mM, 10 mL) solution was added dropwise into the NaBH_4_ (2 mM, 30 mL) under stirring. Subsequently, sodium citrate (340 mM, 500 μL) was added into the mixture and was stirred for additional 10 min. The resulting AgNP_10_ solution was allowed to stand for 2 h at room temperature (RT) and was collected by centrifugation at 12,000 rpm for 15 min. For the synthesis of AgNPs with a diameter of 20 nm (AgNP_20_) [25], AgNO_3_ (25 mM, 500 μL) was quickly added to the mixture (50 mL) of sodium citrate (5 mM) and tannic acid (0.01 mM) under boiling. After 15 min, the mixture was cooled to the RT and stored at 4 °C. The procedure for the synthesis of AgNPs with the diameter of 30 nm (AgNP_30_) was similar with that of AgNR_20_ except that the 0.625 mM tannic acid was used. AgNRs were synthesized by mixing different sizes of AgNPs (1 nM, 500 μL) with signal probes (100 μM, 5 μL) for 48 h. During this time period, NaCl (2 M, 2.5 μL) was added every 4 h until the final concentration of NaCl was 0.1 M. The AgNRs were finally collected by two runs of centrifugation and stored at 4 °C.

Upon synthesis of the above three sizes of AgNPs, their concentrations were determined by the Nanodrop Lite Spectrophotometer (Thermo Scientific, Waltham, MA, USA). The ultraviolet–visible absorption spectra were collected by the UV–vis spectrophotometer (HITACHI, Tokyo, Japan). The morphology and size of AgNPs were further characterized by transmission electron microscope (TEM, JEOL, Tokyo, Japan) with an accelerating voltage of 80 kV. Their hydrodynamic sizes were analyzed by a Zetasizer (Malvern Instruments, Worcestershire, UK).

### 2.4. GNEs Deposition

The bare chip was first cleaned sequentially with acetone, isopropanol, and water for 1 min and blown with nitrogen gas. GNEs were deposited under a constant voltage (0.5 V) in a HAuCl_4_ solution (50 mM in 0.5 M HCl). By adjusting the deposition time (100 s, 150 s, 200 s, 300 s), GNEs with different sizes were obtained. To obtain GNEs with different morphologies, an electrodeposition voltage of 0 V was utilized to obtain a dendric structure. Additionally, electrodeposition with constant current (500 nA) was applied to obtain relatively compact electrode structures. The gold aperture and the nanostructures of the electroplated microelectrodes on the chip were characterized by the scanning electron microscope (SEM, Car Zeiss, Oberkochen, Germany) with electron high tension of 10 kV.

### 2.5. MiR-21 Detection

The capture probe (CP) was diluted with a DNA immobilization buffer (10 mM Tris-HCl, 1 M NaCl, pH 7.4) to 5 μM. The diluted CP was heated at 95 °C for 10 min and cooled at room temperature for 1 h to fully form a stem–loop structure. For surface functionalization, CP (5 μL) was dropped onto the GNEs of the chip and incubate overnight in the dark. MCH (100 μM, 7.5 μL) was then dropped onto GNEs to block excess active sites and remove the non-specific adsorbed probes.

MiR-21 and its mismatched sequences were diluted with the hybridization buffer (10 mM PBS, 1 M NaCl, 40 mM MgCl_2_) prior to miR-21 detection. MiR-21 (5 μL) was dropped onto the chip and incubated at 50 °C for 1 h and then cooled down to room temperature to hybridize with the CP. During this time, the open CP could react with H1 (1 μM, 10 μL) and H2 (1 μM, 10 μL) to trigger HCR at 45 °C in the HCR buffer (10 mM PBS, 1 M NaCl, 20 mM MgCl_2_). To generate electrochemical signals, AgNRs (1 nM, 5 μL) were added and incubated for 1 h. Finally, the chip was washed with PBS before electrochemical measurements.

### 2.6. Electrochemical Measurement

The electrochemical measurements were conducted on the electrochemical workstation (CHI660e, Chinstrument, Shanghai, China) with the Ag/AgCl reference electrode and the Pt auxiliary electrode. The redox signal of AgNRs was collected in KCl (0.3 M) with a scan rate of 0.05 V/s. The electrochemical active surface area (ESCA) for GNEs was measured by cyclic voltammetry in H_2_SO_4_ (10 mM).

*ESCA* was calculated from the reduction peak of GNEs according to the following equation [26]:

(1) $$\mathrm{ECSA}=\int_{PEAK}I\;dV/C$$

where $\int_{PEAK}I\;dV$ is the integration of the reduction peak of GNEs and *C* is a conversion factor of 482 μCcm^−2^.

The probe density was calculated by measuring the electrode surface charge in the presence and absence of [Ru(NH_3_)_6_]^3+^ (50 μM) in 1 mM PBS using chronocoulometry with a pulse width of 0.25 s and scan voltages ranging from 0.2 V to 0.45 V. The chronocoulometric curve was in line with the following equation established by Steel and Cottrell [27]:

(2) $$Q=\frac{2nFA{D_{0}}^{1/2}C_{0}^{*}}{\pi^{1/2}}t^{1/2}+Q_{dl}+nFA\Gamma_{0}$$

where *n* is the number of electrons per molecule for reduction, *F* is the Faraday constant (C/mole), *A* is the electrode area (cm^2^), *D*_0_ is the diffusion coefficient (cm^2^/s), $C_{0}^{*}\;$ is the bulk concentration of [Ru(NH_3_)_6_]^3+^ (mole/cm^3^), *Q_dl_* is the capacitive charge, and *Γ_0_* is the amount of surface-absorbed [Ru(NH_3_)_6_]^3+^ (mole/cm^2^). Chronocoulometric data is plotted as *Q* versus $t^{1/2}$. The intercepts of this curve in the absence and presence of [Ru(NH_3_)_6_]^3+^ are $Q_{dl}$ and $Q_{dl}+nFA\Gamma_{0}$, respectively. $\Gamma_{0}$ can be calculated from the difference between these two intercepts.

Next, the surface density ($\Gamma_{cP}$) can be obtained according to the following equation:

(3) $$\Gamma_{cP}=\Gamma_{0}\left(\frac{z}{m}\right)N_{A}$$

where *z* is the charge of [Ru(NH_3_)_6_]^3+^ and m is the number of bases in CP.

### 2.7. Statistical Analysis

The GraphPad Prism 7.0 was used for statistical analysis. All data were expressed as means ± SEM, and *p* < 0.05 was considered statistically significant. For two group comparison, the student *t*-test was performed; while the one-way ANOVA with Bonferroni post-test was used for multiple comparisons.

## 3. Results and Discussions

### 3.1. Design and Fabrication of HCR-Based e-miRchip

**Scheme 1:**
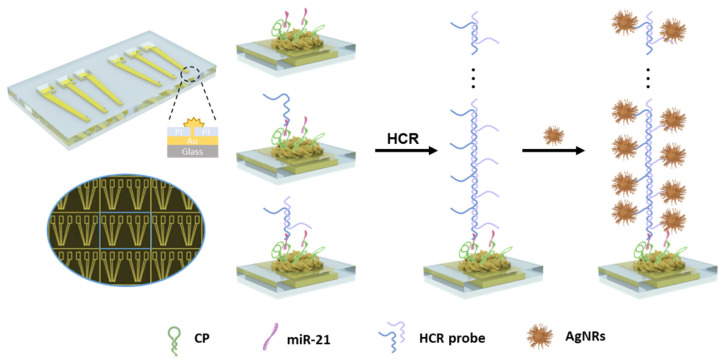
HCR-based e-miRchip for miR-21 detection. The chip was designed with a microaperture array, which allows for the growth of GNEs on top by electrodeposition. MiR-21 was able to open the stem–loop structure of CP immobilized on the GNEs, trigger the HCR and consequently enhance the AgNRs signals.

Previously, our group developed an e-miRchip for direct electrochemical detection of miRNAs [12]. Briefly, this chip was designed by depositing gold patterns (leads and pads) on glass followed by an insulating layer (PI) coating. At the end of each lead, a circular aperture was etched out where a flower-like GNE was electrodeposited (Scheme 1). AgNRs were utilized in this platform to signal and amplify the hybridization of miRNAs on the chip. To further improve the sensitivity of the system, we proposed the use of HCR-based dual amplification strategy in this work to introduce more AgNR binding sites on the e-miRchip. As shown in Scheme 1, the stem–loop CP was first immobilized on the surface of GNEs through Au–S covalent bonds. When the target sequence, miR-21, was added, miR-21 could hybridize with the immobilized CP and open the stem–loop structure. Subsequently, the sequence at the 5′ end of CP was exposed to act as an initiator to initiate the HCR between H1 and H2, ultimately forming an HCR product with a long-repeated DNA helix on the GNEs. Upon the addition of AgNRs, an increased amount of AgNRs were introduced onto the surface of GNEs by hybridizing with the end fragments of H1 and H2. Finally, the enhanced electro-oxidation current of AgNRs corresponding to the concentration of miR-21 was obtained by cyclic voltammetry (CV). With the amplification cascades of HCR and AgNRs on GNEs, sensitive analysis of miRNAs was achieved, providing a promising miRNA detection platform for the point-of-care diagnosis of tumors.

### 3.2. The Optimization of AgNR Sizes and HCR Conditions on Gold Disk Electrode

**Figure 1:**
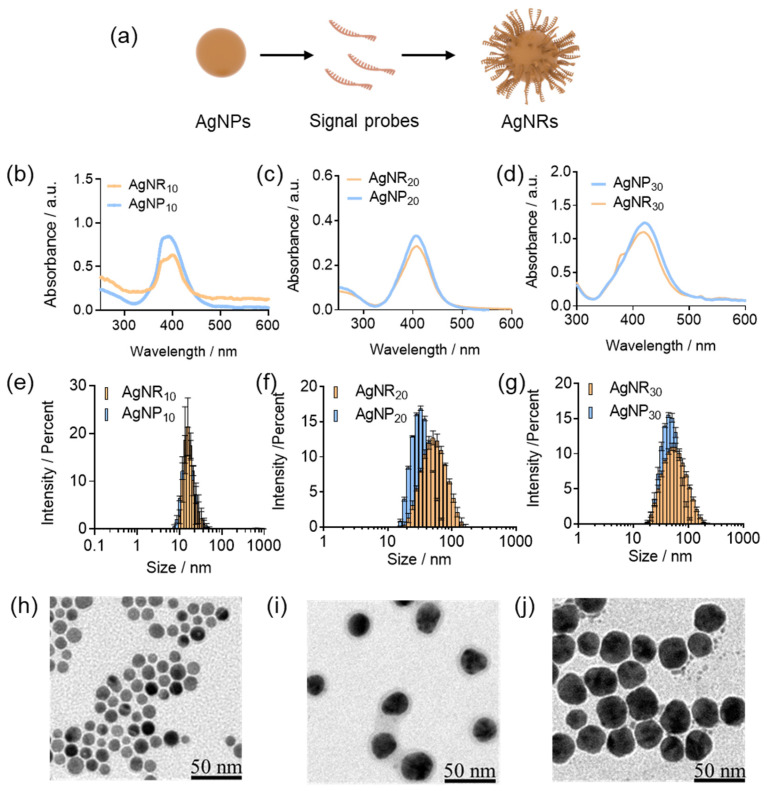
The physiochemical characterization of three sizes of AgNPs and AgNRs. (**a**) A schematic diagram of AgNR fabrication by modifying the AgNPs with signal probes. The UV–vis spectra of the bare AgNPs and AgNRs with diameters of 10 nm (**b**), 20 nm (**c**), and 30 nm (**d**). The DLS measurement of the bare AgNPs and AgNRs with diameters of 10 nm (**e**), 20 nm (**f**), and 30 nm (**g**). The TEM images of AgNR fabricated from three different diameters of the AgNP core of 10 nm (**h**), 20 nm (**i**), and 30 nm (**j**).

To optimize the size for signal amplification, we synthesized and tested the performance of the AgNRs fabricated with three sizes of AgNPs (10 nm, 20 nm, 30 nm). The AgNRs were prepared by overnight incubation of thiol modified signal probes with AgNPs (Figure 1a). The UV–vis spectra of these nanoparticles showed the characteristic absorption peak at 400 nm for AgNP_10_ (Figure 1b), 405 nm for AgNP_20_ (Figure 1c) and 421 nm for AgNP_30_, respectively (Figure 1d); the conjugation of the signal probes on the AgNPs almost did not affect the position of the characteristic peak. Furthermore, DLS was used to further characterize the hydrodynamic size and dispersibility of AgNPs before and after the signal probe modification. Both of the AgNPs and AgNRs exhibited a single characteristic peak, indicating a uniform dispersibility. Due to the purification by membrane filter (0.22 μm), the average hydrodynamic size of AgNR_10_ (17 ± 3 nm) is smaller than that of AgNP_10_ (19 ± 1 nm), but the stable AgNR_10_ in 0.1 M NaCl indicates the successful conjugation of signal probes on AgNP_10_ (Figure 1e). For the other two sizes of AgNRs, their hydrodynamic size was increased compared with the bare AgNPs, in which the hydrodynamic size for AgNR_20_ was increased from 28 ± 3 nm to 44 ± 4 nm (Figure 1f) and the hydrodynamic size for AgNR_30_ was increased from 41 ± 1 nm to 49 ± 0.1 nm (Figure 1g), suggesting the successful conjugation of the signal probes on the AgNPs. From the TEM image, the AgNRs show a sphere shape with the core AgNP size of 11 nm ± 2 nm (AgNR_10_), 21 nm ± 1 nm (AgNR_20_) and 28 nm ± 2 nm (AgNR_30_) (Figure 1h–j). These results demonstrated that three different diameters of the AgNRs (AgNR_10_, AgNR_20_, AgNR_30_) were successfully synthesized by conjugating signal probes onto the AgNP surfaces for subsequent miRNA sensing.

**Figure 2:**
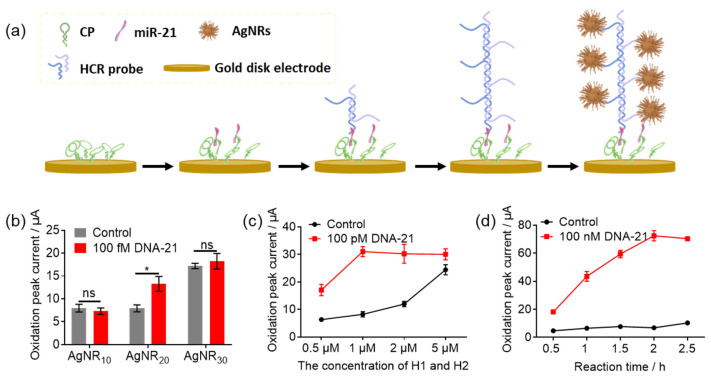
The optimizations of experimental conditions on the disk electrode. (**a**) A schematic diagram of HCR–AgNRs signal amplification strategy performed on the gold disk electrode. (**b**) The comparison of the oxidation peak current of CVs generated by different sizes of AgNRs for DNA-21 (100 fM) detection. (**c**,**d**) The comparison of the oxidation peak current of CVs generated by different HCR conditions for DNA-21 detection, (**c**) different concentration of H1 and H2, (**d**) different reaction time. N = 3 per group, ns: not significant, * *p* < 0.05.

The combination of HCR and the AgNRs-based signal amplification system was first constructed and validated on the gold disk electrode to ensure its effectiveness on electrochemical gene detection. As shown in Figure 2a, the stem–loop CP was first immobilized on the surface of the gold disk electrode. In the presence of DNA-21 (the DNA sequence of miR-21), the stem loop was opened to trigger HCR, and generated an electrochemical signal through AgNR conjugation and the electro-oxidation of Ag (Figure 2a). Based on this gene sensing mechanism, the three synthesized sizes of AgNRs at the same concentration (2 nM) were examined for DNA-21 detection by comparing the oxidation peak current of CV (Figure 2b). We found that the current increased with the increase of the diameter of the AgNRs for the DNA-21 group. However, for the control group, the background currents for AgNR_10_ and AgNR_20_ are similar and much smaller than that of AgNR_30_. Therefore, the highest ratio of signal-to-noise (S/N) is from AgNR_20_ (~1.6), indicating that AgNR_20_ is the optimal size for maximum sensitivity for DNA-21 detection. This may be explained by the following. First, AgNR_20_ had a larger particle size with more Ag atoms, leading to a higher signal response compared to AgNR_10_, whereas the background signal rapidly increases when the AgNR size exceeded 20 nm, leading to a decrease in detection efficiency. Therefore, AgNR_20_ was selected as the optimal particle size. Furthermore, the concentration of HCR probes and reaction time are crucial for the reaction efficiency of HCR [28]. For probe concentration optimization, five concentrations of H1/H2 (0, 0.5 μM, 1 μM, 2 μM, 5 μM) were selected. We found that the oxidation peak current for the DNA-21 detection group increased with the increase of the probe concentration and reached a plateau at 1 μM, while the currents of the control group kept increasing when the concentration of the H1 and H2 probe exceeded 2 μM. This may be due to the non-specific adsorption caused by the high concentrations of the HCR probes. According to the S/N ratio, 1 μM of H1/H2 was chosen as the optimal HCR probe concentration for DNA-21 sensing (Figure 2c). For the HCR reaction time, we found that within the time range of 0.5 h and 2 h, the peak currents for DNA-21 detection rapidly increased and remained unchanged after 2 h, suggesting that the reaction time of 2 h was the optimal reaction time for H1/H2 hybridization to generate a strong DNA-21 detection signal (Figure 2d). Accordingly, for the combination of HCR and AgNR-based gene detection, the optimal size for AgNRs is 20 nm, the optimal HCR probe concentration is 1 μM and the optimal reaction time is 2 h. These optimized conditions were applied for the miRNA detection on the chip in the following sections.

### 3.3. The Verification of HCR-Based e-miRchip in miR-21 Sensing

**Figure 3:**
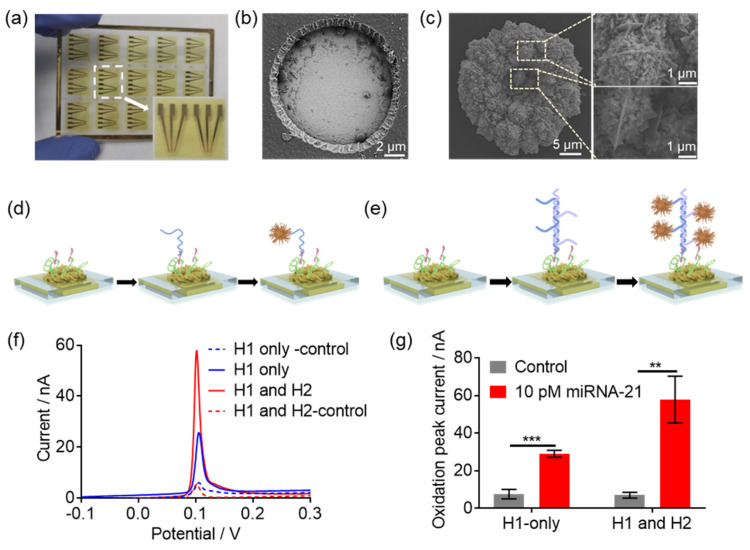
The verification of the HCR–AgNRs signal amplification strategy on the GNEs-electronic chip. (**a**) Photograph of a processed glass with 15 units of chip (**bottom right**). Each chip was designed with six apertures on the tip of the leads for GNE deposition. The SEM of (**b**) the bare aperture and (**c**) electroplated GNEs with the zoom-in high resolution images are labeled with white boxes (**right**). A schematic diagram of HCR-based e-miRchip for miR-21 (10 pM) detection in the presence of (**d**) only H1 and (**e**) both H1 and H2. (**f**) CV scans after AgNRs were added for (**d**,**e**). (**g**) The oxidation peak currents obtained from (**f**). The control group was treated with the hybridization buffer without miR-21. N = 3 per group, ns: not significant, ** *p* < 0.01, *** *p* < 0.001.

Next, we performed the miR-21 detection on the chip with the above-mentioned optimized conditions. As shown in Figure 3a, the electronic chip with 15 sensing units was prepared on glass, which was 1.2 cm × 1 cm in size with six microapertures on the tip of the lead for the simultaneous detection of two different samples with three replicates. The processed microaperture was approximately 11.4 μm in diameter in SEM without obvious damage on the insulation layer (Figure 3b). In order to improve the efficient CP functionalization, a GNE with relatively rough surface was grown from the bottom of the aperture under a small overpotential (0.5 V) (Figure 3c). The diameter of the GNE is measured to be about 31.7 μm with a flower-like morphology. From the zoom-in high resolution images, the nanoflake-like crystal structure could be clearly observed, which increased the surface roughness for more effective hybridization. In addition, we further verified the HCR efficiency on the chip by comparing the detection performance in the presence of only H1 (Figure 3d) and both H1/H2 (Figure 3e). When only H1 was introduced, the miR-21 group showed an increased oxidation current (29.1 μA) due to the AgNRs captured by H1. However, when both H1 and H2 were introduced simultaneously, the oxidation current signal of the miR-21 group significantly increased to about 57.7 μA, which was significantly higher than the corresponding response for the H1 only group (Figure 3f,g). The above observations suggested that only “one-to-one” capture of AgNRs can be achieved in the presence of H1 while “one-to-many” capture of AgNRs occurs for the H1/H2 group. The latter situation triggered the HCR amplification process to produce many HCR products with long-repeated DNA helix structures, thus ultimately achieving “one-to-many” capture of AgNRs, which significantly amplify the electrochemical responses. These results demonstrated the feasibility of HCR-based e-miRchip for miR-21 analysis.

### 3.4. The Effect of GNEs Morphology and Size on miR-21 Detection

**Figure 4:**
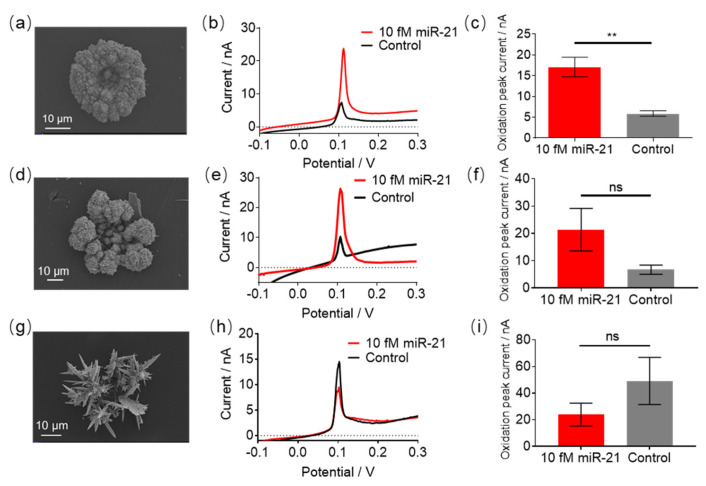
The exploration of the morphology of GNEs for miR-21 detection. (**a**) The SEM image of GNEs deposited under 0.5 V for 200 s using amperometric i-t curve. (**b**) CVs of the electro-oxidation process of GNEs in (**a**) in the presence of miR-21. (**c**) The oxidation peak currents obtained from (**b**). (**d**) The SEM image of GNEs deposited under 500 nA for 80 s using chronopotentiometry. (**e**) CVs of the oxidation process of GNE in (**d**) in the presence of miR-21. (**f**) The oxidation peak currents obtained from (**e**). (**g**) The SEM image of GNEs deposited under 0 V for 30 s using amperometric i-t curve. (**h**) CVs of oxidation process of GNEs in (**g**) in the presence of miR-21. (**i**) The oxidation peak currents obtained from (**h**). The concentration of miR-21 is 10 fM. The dotted line is used to mark the current of 0 nA. N = 3 per group, ns: not significant, ** *p* < 0.01.

It was expected that the morphology of GNEs could be a key factor for the miRNA sensing performance of the chip. Thus, three different morphologies (from more compact structure to relatively loose structure) of GNEs with similar size were electroplated to determine the optimal morphology of GNEs for miR-21 analysis. For the compact flower-like structure deposited under 0.5 V (Figure 4a), the oxidation peak currents of AgNRs in the presence of miR-21 group (10 fM) was significantly higher than that of the control (hybridization buffer only) (Figure 4b) with the S/N of 2.9 (Figure 4c). The relatively loose flower-like structure that represented the more scattered petals was deposited under the constant current of 500 nA (Figure 4d). This structure had a high response for the miR-21 group. However, the response of the miR-21 group is not statistically different from that of the control group (Figure 4e,f). For the dendritic structure under 0 V (Figure 4g), the peak current of the control group was relatively high, making it unable to detect miR-21 (10 fM) (Figure 4h,i). Although this dendritic structure was able to potentially improve the hybridization efficiency, the larger gaps may easily trap the nanoparticles and thus increase the nonspecific adsorption. Taken together, the more compact structure exhibited superior sensitivity with the introduction of AgNRs, confirming the capability of the GNEs to analyze the concentration of miR-21.

**Figure 5:**
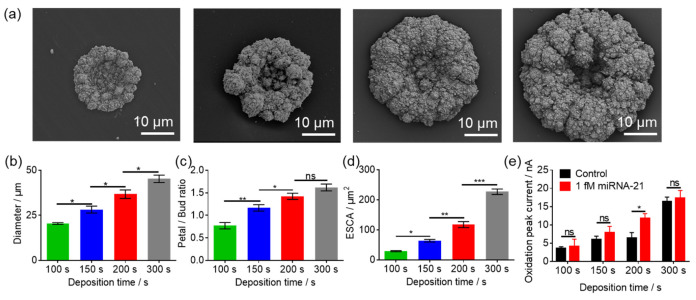
The size optimization of GNEs for miR-21 detection. (**a**) The SEM images of GNEs deposited at 0.5 V for 100 s, 150 s, 200 s and 300 s from left to right. The comparison of (**b**) the diameter, (**c**) the ratio of the diameter of petal to bud, (**d**) the calculated ESCAs of GNEs with different deposition time and (**e**) the comparison of the oxidation peak currents of CVs for GNEs with different deposition times for the detection of 1 fM miR-21. N = 3 per group, ns: not significant, * *p* < 0.05, ** *p* < 0.01, *** *p* < 0.001.

In addition to the morphology, we found that the size of GNEs also affects their performance for miRNA detection in the previous work. Therefore, we further optimized the size of GNEs upon HCR introduction. Four different sizes of GNEs (GNE_100s_, GNE_150s_, GNE_200s_, GNE_300s_) were fabricated by changing the electrodeposition time from 100 s to 300 s. With the different deposition times, we found that all the GNEs exhibited a flower-like morphology with a bud in the middle and petals around it (Figure 5a). As expected, the diameter of GNEs significantly increased when the deposition time increased. The average diameters of GNEs increased from 21 ± 1 μm for GNE_100s_ to 44 ± 3 μm for GNE_300s_ (Figure 5b). Due to the property of hemispherical diffusion of microelectrodes [29], the petal/bud ratio increased in the range of the deposition time of 100 s and 200 s, while the ratio was no longer significant when the deposition time was greater than 200 s, suggesting a fast transport at the edge of GNEs within 200 s (Figure 5c). In addition, the analysis of ECSA also showed that the increase of deposition time not only increased the size but also the roughness of GNEs (Figure 5d). Next, we tested the miRNA detection performance of these four sizes of GNEs for miR-21 analysis (Figure 5e). The result showed that only GNE_200s_ was able to distinguish miR-21 from the control group, while the other sizes of GNEs (GNE_100s_, GNE_150s_, GNE_300s_) showed no significant difference in the presence of miR-21 in comparison with the control. Therefore, the GNE_200s_ represents the optimal GNE size for miRNA sensing.

**Figure 6:**
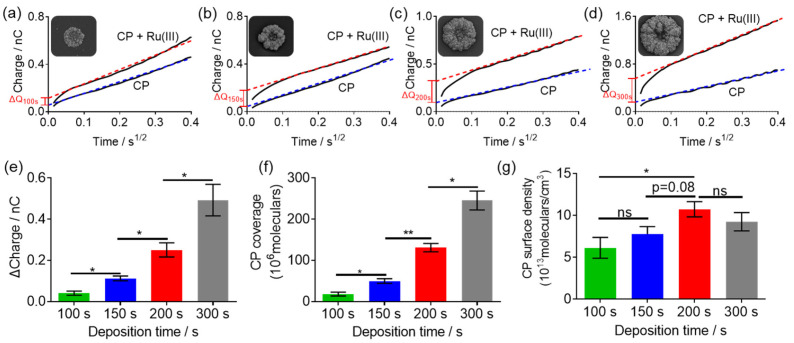
The characterization of the surface charges, CP coverage and CP surface density of the four different sizes of GNEs. (**a**–**d**) The chronocoulometry curves measured in the presence and absence of 50 μM [Ru(NH_3_)_6_]^3+^ in 1 mM PBS for different sizes of GNEs functionalized with CP. (**e**) The calculated Δcharge caused by the introduced CP, (**f**) the calculated CP coverages and (**g**) CP surface density of GNEs with the deposition time from 100 s to 300 s according to the curves in (**a**–**d**). N = 3 per group, ns: not significant, * *p* < 0.05, ** *p* < 0.01.

To explain the above phenomenon, the hybridization efficiency of miRNA and HCR was considered to be a key factor by analyzing the probe density, which was measured through chronocoulometry in the presence and absence of 50 μM [Ru(NH_3_)_6_]^3+^ in 1 mM PBS (Figure 6a–d). According to the intercept of the curves, the charges caused by immobilized CP and the CP coverage for each size of GNEs was obtained. We found that the charges and the coverage of CP increased as the size of GNEs increased, suggesting that the larger the GNEs, the more CP could be immobilized (Figure 6e). However, when taking ECSA into account, the GNE_200s_ exhibited the highest CP surface density (Figure 6g). Studies have shown that a higher probe density usually leads to higher hybridization efficiency for nanostructured electrodes [30]. Accordingly, GNE_100s_ and GNE_150s_ have a low CP density leading to a low hybridization efficiency compared to GNE_200s_. Although there was no significant difference in probe density between GNE_200s_ and GNE_300s_, the large ECSA of GNE_300s_ may result in an increase in nonspecific adsorption of AgNRs, reducing the S/N ratio. Taken together, the deposition time of 200 s was chosen as the optimal condition for GNE fabrication for the subsequent detection of miR-21.

### 3.5. The Detection Performance of the HCR-Based e-miRchip

**Figure 7:**
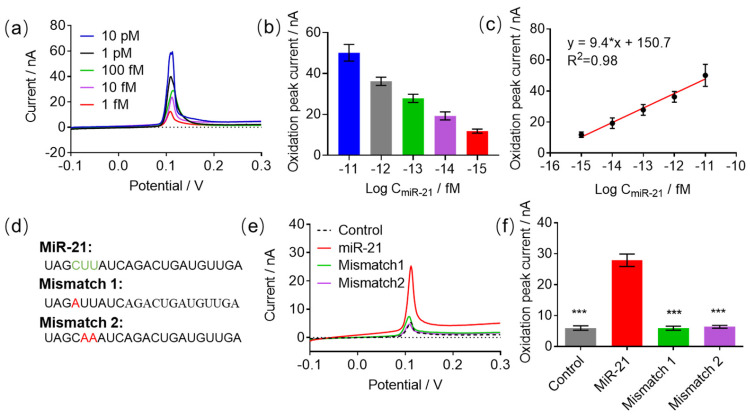
Detection performance of HCR-based e-miRchip on miR-21 sensing. (**a**) CVs for miR-21 detection in the range of concentration from 1 fM to 10 pM. (**b**) The oxidation peak currents obtained from (**a**). (**c**) The linear relationship between the oxidation peak current of CVs and the logarithm of miR-21 concentration. (**d**) A schematic diagram of the comparison among miR-21, single-base mismatched sequence (mismatch1) and double-base mismatched sequence (mismatch2). The bases marked in red represent the mismatched part. (**e**) CVs for miR-21, mismatch1 and mismatch2 sensing. (**f**) The oxidation peak currents obtained from (**e**). The dotted line is used to mark the current of 0 nA. N = 3 per group, *** *p* < 0.001.

The performance of the HCR-based e-miRchip for miRNA detection at the above-mentioned optimized conditions was further validated by the determination of the detection range, the detection limit and the specificity for miR-21 analysis. The gradient diluted miR-21 samples were prepared and added on the chip to collect the CV curves. It was found that the electro-oxidation peak current increased with the increase of miR-21 concentrations (Figure 7a,b). According to the calibration curve ($y=9.4x+150.7,\;\;R^{2}=0.98$), the chip showed a linear relationship within the concentration range from 1 fM to 10 pM of miR-21. Accordingly, the LOD and the limit of quantification (LOQ) for miR-21 analysis were calculated to be 0.9 fM and 5 fM, respectively (Figure 7c), suggesting that the proposed HCR-based e-miRchip was able to qualitatively detect miR-21 in the range of 0.9 fM and 5 fM and quantitatively analyze miR-21 above 5 fM with high accuracy and repeatability. Besides, due to the smaller sample volume (5 μL) used for the HCR-based e-miRchip, the minimum number of miR-21 molecules that has a detectable oxidation current was improved by about 20 times in comparison with the e-miRchip without HCR [12].

In addition, in order to verify the specificity of the constructed system, the HCR-based e-miRchip was used to detect the single-base mismatch (mismatch1) and the double-base mismatch (mismatch2) to compare with the response of the perfect-match miR-21 (Figure 7d). As shown in Figure 7e,f, there is almost no difference between the oxidation currents of the single-base mismatch or double-base mismatch sequence groups and the control group. However, the oxidation current of the miR-21 group had significantly larger signals than both of the single-base mismatch and the double-base mismatch groups, suggesting that the designed stem-loop capture probe containing 15-base pairs was highly stable and was able to distinguish the single-base mismatch sequences, confirming the excellent specificity of the e-miRchip for miRNAs analysis.

It was worth mentioning that the HCR-based e-miRchip showed the advantage of multiplexing due to the fabricated microaperture array among the representative HCR-based gene sensors listed in Table 2. Other methods were mostly based on the bulk electrodes or screen-printed electrodes (SPEs), which could not simultaneously detect multiple samples, leading to a low detection efficiency. Another innovative aspect of this work is the discovery of the optimum morphology for nanostructured electrodes on gene detection. We found that the more compact electrode structure is more effective to reduce the background signal and improve the sensitivity of the electrochemical miRNA detection platform with Ag nanoparticles-based signal probes, which is distinct from previous studies.

## 4. Conclusions

In this work, a new, sensitive and multiplexed e-miRchip platform was developed for miRNA detection. This platform was featured with a dual amplification strategy that combined the HCR-based reaction site amplification mode with the AgNRs-based signal amplification mode. Among three structurally different GNEs with distinct morphology, the flower-like GNEs were found to be the optimal sensing elements for highly sensitive miR-21 detection with the introduction of the AgNRs. This finding provides design guidance for improving the sensitivity of nanostructured microelectrodes with nanosized signal probes. At the optimized conditions, the HCR-based e-miRchip achieved the ultrasensitive detection of miR-21 with a low LOD of 0.9 fM. Furthermore, the minimum number of the detectable miR-21 molecules was reduced about 20 times by the HCR-based site amplification strategy on the e-miRchip in comparison with the absence of HCR. Moreover, this platform was able to effectively distinguish the target miRNA (perfect-match) from the single-base or double-base mismatched sequences. In the future, it is necessary to apply the HCR-based e-miRchip to detect miRNAs in complex biological samples (tissues, blood, etc) to evaluate its clinical applicability. Although such analysis was not performed, encouraged by the excellent sensitivity and specificity of the HCR-based e-miRchip platform developed herein, we believe that it holds great potential as an efficient and low-cost tool and advanced technology for early cancer diagnosis and prognosis.

## Figures and Tables

**Table 1:** The sequences of DNA and RNAs.

DNA/RNA	Sequences (5′-3′)
CP	ATAAGGTTTAGCTTATCAACATCAGTCTGATAAGCTAAACCTCCCC-C_6_-SH
MiR-21	UAGCUUAUCAGACUGAUGUUGA
DNA-21	TAGCTTATCAGACTGATGTTGA
Mismatch1	UAGAUUAUCAGACUGAUGUUGA
Mismatch2	UAGCAAAUCAGACUGAUGUUGA
H1	TAAGCTAAACCTTATGTACATTTCGACGAATAAGGTTAACCACCTCCGATA
H2	ATAAGGTTTAGCTTAAACCTTATTCGTCGAAGGCCACCTCCGATA
Signal probe	SH-C_6_-TTTATCGGAGGTGG

**Table 2:** Comparison of the HCR-based amplification methods for miRNA detection.

HCR-Based Amplification Method	LOD	Sensing Electrode	Multiplexed	Refs.
Tetrahedral DNA/avidin-HRP	10 aM	bulk gold electrode	×	[16]
AgNCs/TAPNR	0.6 fM	bulk gold electrode	×	[17]
AuNPs/AgNPs	2 aM	bulk gold electrode	×	[18]
Au deposition/Exo I	0.9 fM	bulk gold electrode	×	[14]
CdTe QDs/MWCNTs	9 fM	SPE	×	[31]
AgNRs-GNEs	0.9 fM	microaperture array	√	current work

AuNPs: gold nanoparticles; Exo I: exonuclease I; ×: not multiplexed; √: multiplexed.

## Data Availability

The original contributions presented in the study are included in the article, further inquiries can be directed to the corresponding authors.
